# Factors Influencing Sleep Disturbances among Spouse Caregivers of Cancer Patients in Northeast China

**DOI:** 10.1371/journal.pone.0108614

**Published:** 2014-10-02

**Authors:** Quanzhi Zhang, Dazhi Yao, Jinwei Yang, Yuqiu Zhou

**Affiliations:** School of Nursing, Harbin Medical University of Daqing Campus, Daqing, China; University of Pennsylvania, United States of America

## Abstract

**Background:**

In China, spouse caregivers of cancer patients (SCCPs) are involved in all aspects of patient care and experience psychological distress which could result in sleep disturbance and fatigue. However, few studies have explored the differences between SCCPs and the general population, or what factors affect SCCPs' sleep. This study aims to (1) Compare the differences in sleep disturbances and fatigue severity between SCCPs and the age- and gender-matched general population, and (2) Identify selected personal characteristics, including coping style that affect sleep disturbances in SCCPs.

**Methodology/Principal Findings:**

The Stress and Coping Model was used to guide this study. Participants were recruited from the northeast part of China and included 600 people from the general population and 300 SCCPs. Participants completed a socio-demographic form, Fatigue Scale-14, trait Coping Style Questionnaire, and Symptom Checklist-90.

**Results:**

The majority of the participants were middle age, most of whom (78.7%) spent more than 8 hours each day taking care of their spouses. Compared to the general population, the SCCPs experienced significant sleep disturbances with a mean of 7.30 (SD = 1.27), and fatigue severity with a mean of 8.11 (SD = 3.25). Among the selected SCCPs' personal characteristics, current poor health status (β = 0.14, P<0.001), having a spouse under mixed treatment (β = 0.13, p<0.001), and financial burden (β = 0.14, P<0.001) are the significant predictors for sleep disturbances. Positive coping is the predictor for fewer sleep disturbances (*β* = 0.27, *P*<0.001). Those who reported sleep disturbances also experienced higher physical and mental fatigue severity (*P*<0.001).

**Conclusion:**

Intervention to improve coping style in SCCPs is needed. Further research is also needed to explore the other mediators and moderators that regulate sleep disturbance and health outcomes in the SCCPs.

## Introduction

The prevalence of cancer continues to increase globally and remains a leading cause of death worldwide, according to the World Health Organization. Cancer has a large impact on both patients' and caregivers' lives, often resulting in increased levels of tension, pressure, and psychological diseases, and a decline of personal welfare and well-being [1]. As the primary caregivers for cancer patients [2], spouse caregivers of cancer patients (SCCPs) are responsible for the patient's medication, diet, and daily care [3]. Caring for a patient with a life-threatening illness can place a great burden on caregivers who must balance their caregiving tasks with family duties, work roles, and financial responsibilities, that are often altered by their caregiving responsibilities. Previous studies have reported that caregivers experience and share much of the suffering of the patients with advanced cancer for whom they provide care, especially psychological and spiritual distress [4]. In the face of increasing challenges and responsibilities, SCCPs often experience depression. However, little is known about the prevalence and severity of other symptoms of psychological distress in SCCPs.

Sleep disturbance is a significant problem for cancer patients [5] and their family caregivers [6]. In a study by Delgado-Guay et al., most of the caregivers had sleep problems [7]. Interestingly, the prevalence of sleep disturbance experienced by family caregivers of cancer patients is remarkably similar to what is experienced by the patients [8]. Other findings from primarily cross-sectional studies also suggest that many patients [5], [9] and family caregivers [6], [8] report sleep disturbances. Patients [9] and family caregivers [6], [10] report problems on both the initiation and the maintenance of sleep. This findings are not surprising considering the significant amount of stress placed on family caregivers who may be overwhelmed by both their escalating responsibilities to the cancer patient and by the threat of losing a loved one.

It is important to recognize that caregiving involves intense, often negative emotions and stress, particularly when there is uncertainty about what to do and when there is a lack of support [11]. The presence of pre-existing conditions in caregivers may interfere with their ability to assume and fulfill the caregiving role. In addition, caregivers may develop new conditions such as fatigue or the worsening of existing symptoms or conditions during the course of their caregiving activities. Research indicates that caregivers have higher levels of anxiety and depression than is found in the general population or even in cancer patients [12]. Finally, unmitigated symptoms and the demands of caregiving may have an effect on their functional status and quality of life.

Sleep disturbance is linked to negative health outcomes, including impaired cognitive, psychological, or physical functioning, and a lower quality of life [13]. In spite of the absence of findings based on prospective intercorrelations, psychological distress in the form of mood disturbance and anxiety contributed to a dyad's sleep deficits [6], [14]. In three studies, poor sleep was consistently associated with a person's poor psychological well-being [6], whereas in dyads of persons with Alzheimer's disease and their family caregivers, a patient's psychological comorbidity and a caregiver's ineffective coping styles were significant predictors of concurrently manifested sleep disturbance [14]. Among patients with advanced cancer and their family caregivers, patients' body pain and caregiver's global distress were associated with significant sleep disturbance, even though potential interactions were not explored [6]. A caregiver's quality of life might decrease in direct proportion to declines in the patient's functioning and health, and to increases in the patient's need for care and the intensity of the patient's symptoms and distress [15]. This is of great concern not only in terms of the caregivers' mental health, but also because caregivers may be less able to provide adequate support and care to patients.

As patients and caregivers experience the illness together, their emotional reactions, distress, and coping styles also might co-affect their sleep. According to the study of Lee and Hsu, stress contributed to sleep disturbance and depressive mood [16]. Moreover, a patient's symptoms lead to increased caregiving efforts and disrupted caregiver sleep patterns. This, in turn, might hinder management of patient symptoms that influence their sleep. Research has shown that positive or negative coping of the caregiver, influences in coping of the caregiver directly influences the functional status of the person with cancer as well as the caregivers' depression and perceptions of social support [17]. These results suggest that spouse caregivers who utilize more positive, solution-oriented coping styles have less intense sleep disturbance.

The following research questions were addressed in this study: (1) What are the differences in sleep disturbances and fatigue severity between SCCPs and the age- and gender-matched general population? and (2) To what degree are sleep disturbances explained by the SCCP's characteristics (e.g., age, education, financial, health status, etc.) and coping style (negative or positive coping)?

The following hypothesis was tested in this study: (1) spouse caregivers of cancer patients would report higher levels of sleep disturbance and fatigue than the general population; and (2) the selected demographic characteristics (e.g., gender, patient's cancer diagnosis, support by other family members, financial, health status, etc.) and coping styles would be the significant predictor of sleep disturbances in spouse caregivers of cancer patients.

## Materials and Methods

This was a cross-sectional, descriptive, comparative study using multiple self-reported questionnaires to test the above hypotheses regarding the general population and SCCPs.

### Theoretical Framework

The Lazarus and Folkman theoretical model was used as a guide for this study. According to Lazarus and Folkman [18], observed individual differences in stress responses and outcomes are explained theoretically as the result of people's cognitive appraisal and coping style. Contextual factors are elements that make up the history of caregiving and have a potential impact on the stress of SCCPs. In addition, in SCCPs, psychological health can be considered a coping resource that influences the way someone copes with a situation as well as the outcome of these coping efforts. According to Lazarus and Folkman, good psychological health reflects good adaptation outcomes to stressors. Thus, the outcome is the observed SCCP's health status, which includes sleep disturbances and psychological symptoms. This portion of the study focuses on psychological health.

### Study Participants

A total of 300 SCCPs were recruited from two departments in the Fifth Affiliated Hospital of Harbin Medical University and the Da Qing Oilfield General Hospital from September 2011 to January 2012. All SCCPs cared their spouse in the hospital. The inclusion criteria were: (1) a spouse with cancer; (2) a minimum of 6 years of education; and (3) could read and write Chinese. The exclusion criteria included: (1) a history of mental illness; (2) with heart, brain, lung, kidney, or other major disease; and (3) unwilling to participate.

Once identified 300 SCCPs, we recruited the general population in age- and gender-matched with SCCPs. Between May 2012 and July 2012 we recruited 600 general population from community in the Daqing area in China. To be eligible, the general population participants had to: (1) the general population; (2)have spouses not suffering from cancer and major diseases; (3) have no significant incidents in life in the past two weeks; (4) have no history of mental illness; and (5) have a minimum of 6 years of education.

### Ethics Statement

This study was approved by the Medical Ethics Committee of Harbin Medical University. All subjects provided written informed consent.

### Procedures

The Committee on Human Experimentation at Harbin Medical University in Daqing, China approved the ethical standards for this study. Researchers recruited the potential study participants from two research sites (the two sites have the same type of healthcare system), and informed consent was obtained from each study participant.

The study sample included spouse caregivers of cancer patients and the general population. The questionnaires were distributed to 319 spouses of cancer patients. Ten participants dropped out due to reluctance to participate and nine were excluded because the total missing data exceeded 10%. The same questionnaire was distributed to 630 potential study participants in the general population group; however, only 600 study participants were included for the final data analysis (19 uncompleted the questionnaires, and 11 provided no feedback information.)

### Instruments

A researcher-developed demographic form and three psychological soundness instruments were used in this study. The demographic form was used to collect personal and family information, including gender, age, education level, occupation, family income, current health status, caregiving time, and financial burden.

### Symptom Checklist

The Symptom Checklist-90 (SCL-90) [19] is a multidimensional self-report symptom inventory originally designed for use in medical, clinical, and non-clinical samples and based on the Hopkins Symptom Checklist. The inventory is composed of 90 items, each concerned with a distinct symptom of psychopathology. The questionnaire measure the symptoms the study participants experienced in the past seven days. Symptom checklist wherein 1 means not at all, 2 means mild, 3 means moderate, 4 means severe, and 5 means extremely. Psychological symptoms are measured in terms of ten clinical subscales: Somatization (SOM), Obsessive-Compulsive (OBS), Interpersonal Sensitivity (INT), Depression (DEP), Anxiety (ANX), Hostility (HOS), Phobic Anxiety (PHO), Paranoid Ideation (PAR), Psychoticism (PSY), and Sleep (SLE). Any one factor of more than two scores can be considered indicative of psychological problems. In the current study, Cronbach's alphas were 0.97 in SCCPs and 0.98 in the general population group.

### Trait Coping Style Questionnaire

The Trait Coping Style Questionnaire (TCSQ) is a 5-point Likert type scale that consists of 20 items ranging from 1 to 5. 1 means certainly, 2 means generally, 3 means uncertain, 4 means generally not, and 5 means certainly not. Construct validity confirmed two factors in the TCSQ: negative coping (NC) and positive coping (PC). The normal mean of NC is 30.26 (SD, 8.74), PC is 24.42 (SD, 7.14). NC and PC validity and reliability were tested in a Chinese population and reported by Jiang [20], with an 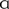 coefficient of 0.69 and 0.70, respectively. In the present study, the NC Cronbach's alphas for the SCCPs and the general population group were 0.74 and 0.76, respectively; the PC Cronbach's alphas for the SCCPs and the general population group were 0.78 and 0.74, respectively.

### Fatigue Scale

The Fatigue Scale-14 (FS-14) [21] was used to measure the study participants' fatigue severity in the past week. This is a 14-item dichotomized survey, and each question is a fatigue-related problem that requires participants to answer. 0 means “no fatigue-related problem”, and 2 means “have fatigue-related problem”. Two components, physical fatigue (8 items) and mental fatigue (6 items), have been identified through factor analysis. Higher scores indicate higher fatigue severity. Its validity and reliability were tested in a Chinese population and reported by Xu [22]; the Cronbach's alpha for the subscale is 0.77. In the present study, the Cronbach's alphas for the spouses of cancer patients and the general population group were 0.75 and 0.78, respectively.

### Methodology and Statistical Analysis

The Statistical Package for Social Sciences (SPSS), version 18.0, was used for data analysis. Univariate summary statistics were examined for all variables. Statistical descriptions were made by use of the mean, standard deviation for continuous variables, and percentage for categorical variables. Independent-sample t tests and Chi-square tests were used to compare differences between groups where appropriate. Hierarchical regression analysis was used to determine which selected predictors significantly account for sleep disturbances in the SCCPs. Regression coefficient (b) and standard deviations (SD) were calculated. All reported P values are two-tailed.

## Results

### Sample Characteristics

The demographics of the SCCP and TGP participants are detailed in Table 1. Chi-square tests confirmed there were no significant differences in age and gender distribution between the two groups. Compared to the general population, the SCCPs experienced significantly more psychological symptoms: positive coping, somatization, depression, anxiety, phobic anxiety, and sleep problems (Table 2).

**Table 1:** Demographic characteristics of SCCPs and TGP.

	SCCPs	TGP	*P value*
	n (%)	n (%)	
Gender			0.33
Men	289 (48.2%)	150 (50.0%)	
Women	311 (51.8%)	150 (50.0%)	
Age, y			0.23
31–40	50 (16.7%)	156 (26%)	
41–50	102 (34.0%)	191 (31.8%)	
51–60	102 (34.0%)	116 (19.4%)	
≥61	46 (15.3%)	137 (22.8%)	
Education			0.98
Primary school	38 (12.7%)	74(12.3%)	
Junior school	90 (30.0%)	189 (31.5%)	
High school	100 (33.3%)	195(32.5%)	
University	72 (24.0%)	142 (23.7%)	
Occupation			0.85
Worker	51(17.0%)	110(18.3%)	
Farmer	30(10.0%)	72(12.0%)	
Civil servant	50(16.7%)	105(17.5%)	
Technologist	38(12.7%)	72(12.0%)	
Service people	36(12.0%)	82(13.7%)	
Retired	67(22.3%)	89(14.8%)	
Other	28(9.3%)	70(11.7%)	
Family income			0.05
1000–2000, yuan	86 (28.7%)	133 (22.2%)	
2000–3000, yuan	146 (48.7%)	334 (55.7%)	
3000–4000, yuan	56 (18.7%)	120 (20.0%)	
≥4000, yuan	12 (4.0%)	13 (2.2%)	
Current health			0.00
Good	558 (93.0%)	210 (70.0%)	
Poor	42 (7.0%)	90 (30.0%)	
Take care time			
3 h	30 (10.0%)	——	
5 h	34 (11.3%)	——	
8 h	52 (17.3%)	——	
10 h	184(61.4%)	——	
Financial burden			
Very large	120(40.0%)	——	
Larger	136(45.3%)	——	
Smaller	28(9.3%)	——	
none	16(5.3%)	——	
Patient's cancer diagnosis			
Lung	93(31.0%)		
Colorectal	41(13.7%)		
Stomach	37(12.3%)		
Breast	81(27.0%)		
Others (Liver, Kidney, Ovarian)	48(16.0%)		
Support by other family members			
Very good	256(85.3%)		
So good	33(11.0%)		
General	11(3.7%)		
Cancer treatment			
Surgery	32(10.7%)		
Chemotherapy	58(19.3%)		
Surgery+Chemotherapy/Radiation	210(70.0%)		

Abbreviations: SCCPs: spouse caregivers of cancer patients; TGP, the general population.

**Table 2:** Differences in Psychological Symptoms between the SCCPs and TGP.

Variable	SCCPs (n = 300)	TGP (n = 600)	Possible score	*T value*	*P value*
	Mean ± SD	Mean ± SD			
Positive Coping	28.23±7.54	30.83±6.90	1–50	4.90	<0.001
Negative coping	24.14±7.77	24.42±6.09	1–50	0.56	0.578
Somatization	19.78±7.62	17.69±6.07	1–60	−4.14	<0.001
Obsessive-Compulsive	18.16±6.09	16.75±5.94	1–50	−3.33	0.001
Interpersonal Sensitivity	14.02±4.74	13.44±4.90	1–45	−1.70	0.090
Depression	22.68±8.54	19.12±6.96	1–65	−6.26	<0.001
Anxiety	15.66±5.97	13.99±5.02	1–50	−4.16	<0.001
Hostility	9.13±3.21	8.91±3.56	1–30	−0.91	0.365
Phobic Anxiety	11.05±3.58	10.26±3.25	1–35	−2.85	0.005
Paranoid Ideation	8.25±2.68	8.25±3.12	1–30	0.00	<0.001
Psychoticism	14.01±4.50	13.37±4.67	1–50	−1.95	0.051
Sleep	7.30±1.27	4.77±2.24	1–35	−5.77	<0.001

Abbreviations: SCCPs, spouse caregivers of cancer patients; TGP, the general population;

A *P*-value of less than 0.05 was considered statistically significant.

### Comparison of the Differences of Selected Variables between Spouse Caregivers and the General Population

Independent *t*-tests were used to compare the differences of sleep disturbances and fatigue severity between the SCCPs and the general population. Compared to the general population, the spouse caregiver group reported statistically significantly higher sleep disturbances. The mean (SD) sleep disturbance of the SCCPs and the general population was 7.30 (1.27), and 4.77 (2.24), p<0.001 respectively. The SCCPs had greater total, physical, and mental fatigue than the general population with a mean of 8.11 (SD, 3.25), 4.99 (SD, 2.43), and 3.12 (SD, 1.66), respectively (Table 3).

**Table 3:** Sleep scale and fatigue level comparison between SCCPs with TGP.

Variable	SCCPs (n = 300)	TGP (n = 600)	*P value*
	Mean ± SD	Mean ± SD	
Sleep	7.30±1.27	4.77±2.24	<0.001
Physical Fatigue	4.99±2.43	4.36±2.42	<0.001
Mental Fatigue	3.12±1.66	2.75±1.65	<0.001
Total Fatigue	8.11±3.25	7.11±3.47	<0.001

Abbreviations: SCCPs, spouse caregivers of cancer patients; TGP, the general population.

Table 4 summarizes the results of the hierarchical multiple regressions performed. Results revealed significant effects on sleep disturbances. The significant predictors for sleep disturbances include: current poor health status (β* = *0.14, p<0.001), having a spouse under mixed treatment (β = 0.13, p<0.001), and financial burden (β = 0.14, p<0.001). Positive coping is the predictor for less sleep disturbances (β = 0.27, p<0.001). Those who reported more sleep disturbances also experienced higher physical and mental fatigue severity (p<0.001).

**Table 4:** Predictors for Sleep Disturbances Severity among the SCCPs.

	B	SE	*β*	t
Current poor health status	0.86	0.011	0.139	78.16^c^
Spouse under mixed treatment	−0.80	0.011	−0.128	−72.11^c^
Financial burden	0.80	0.010	0.137	77.06^c^
Positive Coping	−0.10	0.001	−0.267	−153.27^c^

Abbreviations: SCCPs, spouse caregivers of cancer patients.

c *P*<0.001 (compare with pre-rehabilitation value).

## Discussion

This study evaluated the differences in the occurrence and severity of sleep disturbances between SCCPs and the general population, and identified how selected personal characteristics and coping style affect sleep in SCCPs. As the number of patients with cancer and the number of cancer survivors of all ages increase, and as cancer survivors live longer, the role of caregivers grows as well. SCCPs are assuming more and more responsibilities for the care of their loved ones as healthcare delivery grows increasingly more complex and outpatient focused. It is important to recognize that caregiving involves intense, often negative emotions (e.g., somatization, obsessive-compulsive, depression, and anxiety) and stress [11]. Caregivers provide more than half the care needed by patients with cancer, although the formal healthcare system rarely prepares them for that role [23]. Consequently, caregiving has a substantial impact on SCCPs' physical and mental well-being and can negatively influence both patient and caregiver health outcomes [24]. A recent study found that caregivers' negative emotional state, cognitive and physical impairment (including sleep and fatigue), and low literacy were serious impediments to their effective management of patients' medications [25]. A large study of family caregivers (N = 1,662) found that their unmet needs for providing symptom management and other types of support negatively affected the quality of their caregiving at the patients' end of life [26]. Other researchers reported that spouse caregivers who had high emotional distress early in the course of their spouses' illnesses had a significant negative effect on the adjustment of these cancer patients 1 year later [27].

### Sleep Disturbances of SCCPs

Sleep is a basic human need. High-quality sleep has a restorative, protective, and energy-conserving function that enables people to continue their daily living activities [28]. In the present study, compared to the general population, the SCCPs experienced significantly more sleep disturbances (*P*<0.001). Fatigue was reported as another symptom in some SCCPs (*P*<0.001). A study by Carter and Chang [29] found that 95% of the caregivers experienced moderate to severe sleep disturbances as measured by subscales that assessed sleep quality, duration, efficiency, disturbances, and daytime function. A more recent study by Fletcher et al. [30] reported that sleep disturbance could be a factor in identifying cancer patients' family caregivers at highest risk for sustained fatigue trajectories.

### Reasons for Sleep Disturbance

#### Poor health

In the present study, “current poor health status” was one cause of sleep disturbance in SCCPs. In the sample, nearly half of the SCCPs had at least one symptom, although the illness accounted for only a small percentage of the overall reasons for sleep disturbance. The caregiving situation itself may be associated with negative health effects [31], [32] that can adversely impact sleep. Therefore, an SCCP's caregiving role throughout the cancer trajectory may be interrupted by self-care activities. One study found an association between the demands of caregiving and the SCCPs' risk of morbidity because of their caregiver role [33]. Several other studies also attributed caregiving demands to a decline in personal health status. For example, the frequently cited Caregiver Health Effects Study [34] found that spousal caregivers who reported caregiving strain had a 63% greater risk of death 4 years later than non-caregivers who were matched on demographics and physical health status. These results can be used to classify caregivers who experience the stressors associated with caring for someone with a chronic illness and who have chronic medical conditions themselves.

#### Mixed treatment and financial burden

The second reason for sleep disturbance is “patients under mixed treatment.” The majority of cancer patients undergo surgery, radiotherapy, chemotherapy, and other treatment processes and accumulates medical expenses that result in financial hardship and a long-term increase of psychological symptoms, which could lead to sleep disturbance. As patient needs increase, primary caregivers are expected to provide intensive care, which allows only for minimal periods of rest and sleep [35], [36]. One study estimated that family caregivers of patients with cancer averaged 8.3 hours per day of caregiving over 13.7 months [37]. In the present study, the majority (78.7%) of SCCPs spent more than 8 hours each day taking care of their spouse.

Several studies have focused on the sleep of caregivers, including studies that were related to sleep quality. As demonstrated in one study [38], the caregivers of cancer patients had little time to rest and slept inadequately, while another study [39] showed that the quality of sleep of 95% of caregivers of cancer patients was poor. Two meta-analyses [40] examined the relationship between the psychological distress of patients with cancer and their spouse caregivers who were also their primary caregivers. This study found that the patients' and the caregivers' responses to cancer were interdependent and each person affected the other's level of emotional well-being.

An additional reason for sleep disturbance was “job stress.” Regular employment and insurance are essential to one's ability to pay for expenses during the cancer trajectory. However, the stress levels of work can contribute to the burdens felt by the caregivers. In turn, their sleep architecture can be affected. In one study [41], employed caregivers of cancer patients who were also responsible for taking care of their children had high levels of stress. The authors of this study concluded that caregivers taking on more than one social and familial role had increased stress levels.

The China Social Security Institution does not pay for all medications; thus, some cancer patients must pay for it on their own. This problem was thought to cause psychological distress in patients and their spouses, which would lead to sleep disturbances. One study showed [18] that financial worries were among the top three problems that caregivers encountered. In another study, economic burden was calculated from the accumulated value of a caregiver's time providing care, value of lost employment, and out-of-pocket expenditures [42]. The current study found that a sense of fatigue was significantly higher for SCCPs with a family income of 1000–2000 yuan than for SCCPs with a family income of 2000 yuan or more. Time costs and out-of-pocket expenditures clearly demonstrate the sizeable financial burden that caregivers endure, often with limited resources.

#### Coping style

In the present study, findings support general coping research in that coping style is associated with psychological functioning. For example, Kershaw *et al.* [37] found that avoidance coping in caregivers of breast cancer patients was associated with lower mental quality of life to a larger degree than any other strategy examined. Ptacek *et al.* [38] found that problem-focused coping is associated with higher marital satisfaction and emotion-focused coping is associated with lower marital satisfaction.

Different cognitive coping styles are important factors in individual environmental adaptability and psychological health. In our study, positive coping styles can reduce the level of stress response, thus affecting the relationship between stress and mood disorders. Positive ways of coping result in better psychological adjustment, while negative coping styles are maladaptive [39]. Another study [40] have found active adjustment to be more often associated with positive coping styles, whereas more emotion-focused or negative coping styles are more often associated with higher anxiety and depression at different times during treatment [42].

Results from the current study revealed that SCCPs experienced stress, fatigue, and sleep disturbances. It is essential to have well-prepared SCCPs to promote their well-being.

### Limitations

There are several limitations to our study. First, the study sample was recruited in the northeast part of China and was not representative of all of China. Second, although we investigated common predictors among two different groups, unique predictors and outcomes for each group should be further investigated. Third, in this study we use the sleep disturbances subscale from the Symptom Checklist-90 to measure sleep. The exclusive questionnaire of sleep should be used in future studies. Fourth, The level of severity of cancer in patients (such as stage, daily activity level) should be provided, because sleep disturbances might be associate with the care contents, such as changing tubes, feeding, changing position, wound care. Finally, the two groups were recruited from different settings (which resulted in groups with different demographic characteristics, such as family income, lifestyle, and current health) and with somewhat different inclusion criteria, and the data from each group were assessed at different times.

### Conclusion

This research reveals that most of the SCCPs had poor sleep quality. The SCCPs also expressed many reasons for their sleep disturbances. These reasons were generally related to their current poor health status (e.g., having a specific illness of their own), a spouse under mixed treatment, financial burden, and coping style. To prevent negative outcomes, nurses should be aware of the sleep disturbances of SCCPs in addition to the problems of cancer patients during the caregiving process.

### Directions for Future Research

Currently, caregiver research is not a national priority, but it needs to have equal standing with other areas of medical research. Future research needs to (1) examine how preparation of patients' spouses for their caregiving role affects both patient and caregiver outcomes; (2) identify a cumulative set of risk factors to determine which caregivers are at risk for distress and then examine how caregivers' risk status affects patients' and caregivers' long-term outcomes and the interventions that are most effective for reducing distress in these caregivers; (3) examine caregivers' physical health status and pre-existing co-morbidities, and determine how their health status changes as caregivers' assume or relinquish their role; and (4) identify cost-effective interventions for SCCPs and determine how they affect the overall cost of patient care and use of services.
